# Electrocardiographic characteristics of idiopathic ventricular arrhythmias based on anatomy

**DOI:** 10.1111/anec.12782

**Published:** 2020-06-27

**Authors:** Yulong Xiong, Hongling Zhu

**Affiliations:** ^1^ Tongji Medical College Huazhong University of Science and Technology Wuhan Hubei China; ^2^ Department of Cardiology Tongji Hospital Tongji Medical College Huazhong University of Science and Technology Wuhan Hubei China

**Keywords:** cardiac anatomy, ECG, idiopathic ventricular arrhythmias

## Abstract

Idiopathic ventricular arrhythmia (IVA) is a term used to describe a spectrum of ventricular arrhythmia without structural heart disease (SHD). IVAs contain premature ventricular contractions (PVCs), nonsustained monomorphic ventricular tachycardia (VT), and sustained VT. Electrocardiography is a fundamental and important tool to diagnose and localize IVAs. More detailed, IVAs originating from different origins exhibit characterized ECGs due to their specific anatomic backgrounds. As catheter ablation becomes widely used to eliminate these arrhythmias, its high success rate is based on accurate localization of their origins. Therefore, these ECG characteristics show great importance for precise localization of their origins and subsequently successful ablation. This review aims to sum up ECG characteristics of IVAs based on anatomy and give brief introduction of mechanisms and treatment of IVAs.

## 1 INTRODUCTION

Idiopathic ventricular arrhythmias (IVAs) account for approximately 10% of patients with ventricular tachycardia (VT) (Joshi & Wilber, 2005). Most IVAs originate from outflow tract (OT) of either ventricle (Stevenson & Soejima, 2007), but other sources including fascicles, papillary muscles, mitral and tricuspid annuli, and epicardium are of great importance as well. Characteristic ECGs illustrate IVAs from different sites of origin owing to specific anatomy and guide catheter ablation, which has become a widely used tool to eliminate ventricular arrhythmias (VAs). Therefore, it is essential to study the characteristics of the surface ECGs of IVAs according to their anatomy to choose the optimal ablation method and improve the success rates. In this review, we will summarize the salient ECG characteristics of IVAs (Table I) based on anatomy.

**Table I:** ECG Characteristics of Idiopathic Ventricular Arrhythmias

Sites of Origin	BBB	Axis	Precordial Transition	Other ECG Features
Right Ventricle				
RVOT				
Septal	LBBB	Inferior	≥V3	Narrower QRS complexes with higher R waves in inferior leads
Free‐wall	LBBB	Inferior	≥V4	Broader QRS complexes with smaller R waves and Notchings in inferior leads
Tricuspid Annulus				
Septal	LBBB	Superior deviated	≤V3	QS pattern in lead V1
Free‐wall	LBBB		>V3	Longer QRS duration; Nothcings in limb leads
Moderator Band	LBBB	Left Superior	>V4	Relatively narrow QRS complexes
Papillary Muscles				
Anterior/Posterior PAP	LBBB	Superior	>V4	Wider QRS complex with notchted precordial leads
Septal PAP	LBBB	Inferior	≤V4	
Parahisian	LBBB	Left inferior	V2‐V3	Narrower QRS duration in inferior leads and smaller R‐wave index
RVOT TA junction	LBBB	Inferior	V2‐V4	Flat QRS complex in lead aVL; deep negative wave in lead aVR
Left Ventricle				
LVOT				
ASV				
LCC	LBBB	Inferior	≤V2	Significant R‐wave or multiphasic pattern (“m” or “w” morphology) in lead V1; greater R‐wave amplitude ratio
RCC	LBBB	Inferior	≤V3	High R‐wave amplitude in lead I
RCC‐LCC junction	LBBB	Inferior	V3	QS morphology in lead V1 with notching on the downward deflection
NCC	LBBB	Inferior	V2‐V3	Narrower QRS duration, smaller III/II ratio
AMC	LBBB/RBBB	Inferior	V1/None	qR pattern in lead V1
Septal‐parahisian	LBBB	Left inferior	V2‐V3	Negative QRS polarity in lead III and positive QRS polarity in lead aVL
Epicardium				
Summit region	LBBB/RBBB	Inferior	None/<V1	MDI > 0.55; pseudodelta wave ≥ 34ms IDT ≥ 0.55; RS complex duration ≥ 121ms
Cardiac crux	LBBB/RBBB	Superior	Early	
Mitral Annulus				
Anterolateral	RBBB	Inferior	V1/V1‐V2	Notching in the late phase of the QRS complex in inferior leads and longer QRS duration
Posterior	RBBB	Superior	V1/V1‐V2	
Papillary muscles				
APM	RBBB	Right inferior	<V1	qR or qr pattern in lead aVR and rS pattern in lead V6
PPM	RBBB	Superior	<V1	Small mean III/II ratio
Fascicles				
Left posterior	RBBB	Superior	Early	Loss of late precordial R waves with more apical exits
Left anterior	RBBB	Right	None	
Upper septal	Normal or IRBBB	Normal or Right	V3	Narrow QRS complex; Q wave in inferior leads and/or an S wave in lead I and/or aVL

## 2 RELEVANT ANATOMY

The right ventricle (RV) could be divided into three components (Goor & Lillehei, 1975): the inlet; the apical trabecular myocardium; and the outlet, also called right ventricular outflow tract (RVOT). The RVOT is a tube‐shaped structure wrapping anteriorly around the left ventricular outflow tract (LVOT) and the aortic root in a posterior and leftward direction (McGuire et al., 1996). More detailed, the RVOT incorporates two opposing crescentic aspects: the anterolateral or “free‐wall” surface and the posteromedial or “septal” surface.

It has been validated that the ventricular myocardium extends into the great arteries in variable patterns and distances, and become potential substrates for the IVAs (Anderson, 2000; Asirvatham, 2009; Cabrera & Sanchez‐Quintana, 2013; Gami et al., 2011; Hasdemir et al., 2007; Talreja et al., 2001). They are reported to exist in the pulmonary artery among nearly 90% individuals and shown to be a more frequent source of IVAs (nearly 50% of RVOT‐type arrhythmias) than previously recognized (Liu et al., 2014). In aortic sinuses, the left coronary cusp (LCC) is the most frequent site of origin of IVAs arising from LVOT, followed by the right coronary cusp(RCC), the RCC‐LCC commissure, and finally the noncoronary cusp(NCC) (Yamada, McElderry, Doppalapudi, Murakami, et al., 2008).

The tricuspid valve incorporates three leaflets located septally, anterosuperiorly, and inferiorly. Each leaflet is attached to the corresponding papillary muscles(PAP) with tendious cords (Muresian, 2016).. The moderator band(MB) is a part of septomarginal trabeculation which extends from septum to free wall, encompassing RV Purkinje fibers, and supporting the anterior papillary muscle (Sadek et al., 2015). Additionally, some rare sites in the RV including parahisian region, the RV apex, and RVOT TA junction can also be observed as sites of origin of IVAs in some patients (Ho & Nihoyannopoulos, 2006; Lu et al., 2016; Satish, Yeh, Wen, & Wang, 2005).

The left ventricle incorporates three components as well. However, the LV sites of origin are more complicated, including LVOT, mitral annulus (MA), papillary muscles, fascicles, and epicardial regions. The LVOT can be further divided into supravalvular (aortic sinus of Valsalva), infravalvular (AMC and septal‐parahisian region), and epicardial (the LV summit) portions.

The mitral valve has two leaflets positioned anteriorly and posteriorly attaching to the corresponding papillary muscles(Muresian, 2009). The aortic and mitral valves attach to an elliptical opening at the base of the LV known as the LV ostium (Lerman, 2015). Between them is a fibrous area called the aorto‐mitral continuity (AMC). Despite of its fibrous components, ventricular arrhythmias arising from AMC have been reported and the remnants of the conduction system during embryonic development might explain this uncommon arrhythmia (Mizobuchi & Enjoji, 2015; Szili‐Torok, van Malderen, & de Groot, 2012).

Two main epicardial regions are associated with IVA, namely the LV summit and the cardiac crux. The LV summit was first defined by (McAlpine, 2012) and refers to the most superior portion of the LV epicardium, abutting the LCC. It is a triangular region demarcated by the left anterior descending (LAD) and the circumflex coronary (LCx) arteries and transected by the great cardiac vein (GVC) into two regions. The inferior one is accessible for ablation whereas the superior one is not. Another epicardial origin is cardiac crux in the posteroseptal region, which has a pyramidal space representing the confluence of all four cardiac chambers and the coronary sinus in their nearest proximity. It is further divided into the basal and apical crux with different treatments (Kawamura et al., 2014).

However, cardiac anatomy is affected by many factors with anatomical and functional variations. Some cardiac structures have congenital discrepancies among individuals. For example, there exists an apparent variability in the relationship among the components of the tricuspid valve(Tretter, Sarwark, Anderson, & Spicer, 2016). And the papillary muscles vary markedly in their numbers, shapes, and patterns in both ventricles(Saha & Roy, 2018; Xanthos, Dalivigkas, & Ekmektzoglou, 2011). Such congenital variations exist in the conduction system as well, which may partially account for the rhythm abnormalities after transcatheter surgery(Saadi et al., 2018). Besides, the aging process is of great importance and should be taken into account in clinical practice. Age‐related gross changes include a proximal bulge in the interventricular septum and a rightward‐shifted ascending aorta, which may lead to a narrowing of the LVOT(D. Goor, Lillehei, & Edwards, 1969). Although habitual exercise is shown to oppose many age‐related alterations(Ferrari, Radaelli, & Centola, 2003; Hunt, Farquhar, & Taylor, 2001), long‐term endurance exercise training may cause right ventricular dilatation, diastolic dysfunction, and cardiac fibrosis, which may provide a substrate for RVOT free‐wall tachycardia(Gülan et al., 2019). In conclusion, cardiac variations are vital for accurate diagnosis and surgical procedures.

## 3 MECHANISMS OF IDIOPATHIC VENTRICULAR ARRHYTHMIA

Mechanisms behind IVAs are related to anatomical structures as well. Both ROVT and LVOT arrhythmias are shown to share similar electrophysiological and pharmacological properties, including sensitivity to adenosine. This suggests, despite of different sites of origin, a common arrhythmogenic mechanism consistent with catecholamine‐induced, cAMP‐mediated delayed after depolarization, and triggered activity. Therefore, these arrhythmias should be considered as a single entity and classified together as “outflow tract arrhythmias” (Daniels et al., 2006; Iwai et al., 2006; Maury, Rollin, Mondoly, & Duparc, 2015).

Intravenous isoproterenol infusion could induce IVAs originating from tricuspid or mitral annulus whereas programmed ventricular stimulation could not.(Kumagai et al., 2005; Tada et al., 2007) This indicates that the underlying mechanisms for these IVAs might be triggered activity or automaticity. Similar to AMC, remnants of atrioventricular conduction system might be important in genesis of the nonreentrant MA VA(Tada et al., 2005).

The fascicular VT, known as “verapamil‐sensitive” VT given its tendency to slow or terminate with intravenous verapamil, is a reentrant tachycardia involving the left fascicular Purkinje system (Talib et al., 2015; Tsuchiya, Okumura, Honda, Iwasa, & Ashikaga, 2001). It is caused by a reentrant circuit incorporating the Purkinje system with an excitable gap and a slow conduction area (Nogami et al., 2000; Tsuchiya et al., 2001). Besides, focal Purkinje VT, which is observed in both structurally normal hearts and ischemic heart disease (Gonzalez et al., 1994; Lopera et al., 2004; Rodriguez, Smeets, Timmermans, Trappe, & Wellens, 1996), is found to be “propranolol‐sensitive” with abnormal automaticity as the underlying mechanism (Nogami, 2011).

## 4 ECG CHARACTERISTICS OF IDIOPATHIC VENTRICULAR ARRHYTHMIA

### 4.1 General ECG characteristics

**Figure 1:**
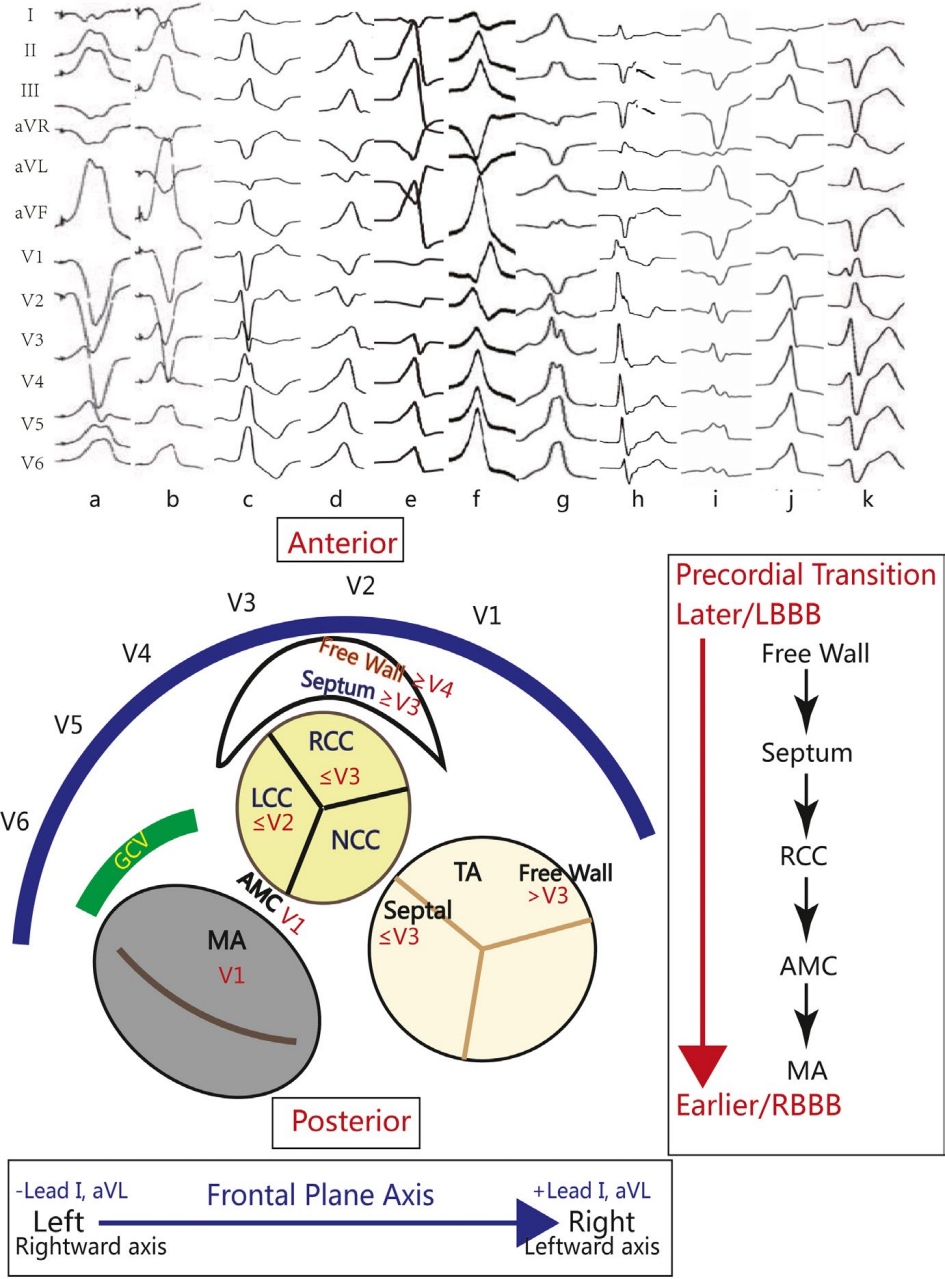
An simplified schema for understanding the general ECG morphology (precordial transition and frontal plane axis) is shown. Surface 12‐lead ECG of IVAs originating from the RVOT free‐wall(a), RVOT septum(b), RCC(c), RCC‐LCC commissure(d), LCC(e), AMC(f), septal‐parahisian region(g), posterior MA(h), posterior septum of TA(i), the summit region(j), and the left posterior fascicle(k) are shown above

The ECG patterns of IVAs are determined by its site of origin based on anatomy. Although variabilities of ECG interpretation among individuals exist, there are some general features to help localizing the IVAs. Firstly, the anterior structures tend to have a later precordial transition while the posterior sites of origin possess an earlier precordial transition. Thus, RVOT VT and the anterior sites of LVOT (e.g., RCC) mostly have an LBBB configuration. As the site of origin shifts more posteriorly from RCC, a transformation from LBBB to RBBB ensues (Hutchinson & Garcia, 2013) (Figure 1). Secondly, for the frontal plane QRS axis, both horizontal and vertical dimensions should be taken into account. The bipolar limb lead I represents the horizontal dimension most efficiently and leads II/aVL, III/aVR are also useful due to their net horizontal vectors (Hutchinson & Garcia, 2013). Origins that are relatively leftward have negative polarity (QS, Qr or rS) in leads I and aVL because of their rightward axis. Therefore, R waves become increasingly predominant in these leads in patients with more rightward sites of origins (Haqqani, Morton, & Kalman, 2009; Lerman, 2015; Yamauchi et al., 2005) (Figure 1). The vertical dimension is reflected by the inferior leads (leads II, III, and aVF). An inferior axis suggests an origin in the superior aspect of the ventricle, whereas a superior axis indicates an origin in the inferior surface (Yamada, 2016). For example, the mean QRS axis of OT‐VAs is inferiorly directed with rare exception. However, the amplitude of the vector diminishes as the site descends within the OT.

### 4.2 Right Ventricle

#### 4.2.1 RVOT

IVAs from the ROVT are most commonly seen and account for nearly 75% of all OT VA cases.(Park, Kim, & Marchlinski, 2012) The free‐wall sites of origin has been previously reported in 20%‐30% of patients with RVOT VT but can vary from 9% to 34% (Joshi & Wilber, 2005; Tada et al., 2004). The common ECG features of the RVOT VA include LBBB configuration and an inferiorly directed frontal plane QRS axis which represents deeply negative QS complexes in lead aVL, aVR and positive polarities in the inferior leads (Park et al., 2012). The septal sites of origin tend to have narrower QRS complexes (<150msec) with higher R waves in inferior leads. In contrast, the free‐wall locations have broader QRS complexes (≥150msec) and smaller R waves in inferior leads that are characterized by “notching” (Dixit, Gerstenfeld, Callans, & Marchlinski, 2003; Yu et al., 2009). Given the relative position of the free‐wall and septal surfaces, the free‐wall surface has a later precordial transition (V4 or V5) while the septal surface has earlier precordial transition (V3 or V4). Importantly, a combination of both later precordial transition and notching in inferior leads shows more accurate predictions.

The vast majority of RVOT VA, both septal and free‐wall, originates from myocardium within the first 1–2 cm beneath the pulmonary valve. (Joshi & Wilber, 2005) Therefore, it is important to identify the leftward or rightward and the superior or inferior position in the RVOT by methods described above. Generally, for the leftward portion in the RVOT, the ECG patterns manifest negative or flat in lead I and more negative in lead aVL compared with aVR (aVR/aVL > 1). On the contrary, the rightward aspects show a positive lead I and more negative in lead aVR than aVL (aVR/aVL ≥ 1). VAs originating from the superior sites appear greater magnitude in the inferior leads (II, III, aVF) and higher initial r‐wave amplitude in lead V1 and V2 (V1 ≥ 0.15mV and V2 ≥ 0.3mV) than the inferior sites (Yu et al., 2009).

The myocardial extension into the pulmonary artery might provide substrates for 4% of the IVAs (Tada et al., 2008). The sites within the pulmonary artery are more leftward and superior than the RVOT. However, the ECG characteristics of the PA VAs remain unclear with some contradictory trial results (Liu et al., 2014; Tada et al., 2008) and a negative finding in a meta‐analysis (Wang, Zhang, Hong, & Huang, 2017).

#### 4.2.2 Tricuspid Annulus

The tricuspid annulus(TA) accounts for about 8% of the IVAs and is further divided into the “septal” and the “free‐wall” portion for precise location (74% from the septal aspect) (Tada et al., 2007). Because the tricuspid valve is located on the right anterior side of the heart and right inferior side of the RVOT, the mean QRS vector of TA‐VA is directed more leftward and superiorly than the QRS vector of the RVOT VA. Therefore, all IVAs originating from the tricuspid annulus exhibit an LBBB pattern and a greater R or r wave in lead I than the RVOT VA. Most of the QRS polarities appear positive in lead aVL and negative in the inferior leads. These findings are helpful for distinguishing the TA‐VA from the RVOT VA. To identify the precise location of the TA‐VA depends on other ECG characteristics such as presence of QRS “notching” and precordial transition (Sato et al., 2019; Tada et al., 2007).

#### 4.2.3 Right Ventricular Papillary Muscles

Both left and right ventricular papillary muscles could give rise to IVAs with an incidence ranging from 4% to 12% while the reported cases involving RV papillary muscles are scarce (Naksuk, Kapa, & Asirvatham, 2016). Among the three groups of RV papillary muscles, the septal papillary muscle, arising from the RVOT and usually referred as the conus papillary muscle(CPM), seems to be the most common origin of IVAs (Crawford et al., 2010). Therefore, IVAs originating from this structure might be confused with arrhythmias originating from RVOT. However, patients with CPM‐genic arrhythmia sometimes show pleomorphic ECG morphologies. This may be explained by the fact that the CPM is so proximal to the His and right bundle that the Punkinje fibers origin may account for the polymorphic arrhythmia (Hai, Desimone, Vaidya, & Asirvatham, 2014). In general, arrhythmias originating from the RV papillary muscles had an rS/QS pattern in lead V1 and display an LBBB morphology. The mean QRS width appears greater(>160ms) and notchings in the precordial leads are mostly present. IVAs originating from the anterior or posterior PAP are more likely to have a later R‐wave transition(>V4) and a superior axis compared with those from the CPM, which more often have an earlier R‐wave transition(≤V4) and an inferior axis. Such discrepancy corresponds with more apical insertion of both anterior and posterior PAP and more basal insertion of the CPM(Crawford et al., 2010).

#### 4.2.4 The moderator band

The moderator band(MB) has only been recognized recently as a site of origin of IVAs and a source of triggers of ventricular fibrillation(VF) (Anter, Buxton, Silverstein, & Josephson, 2013). Relevant information about the prevalence, ECG features, and results of ablation of MB VA is limited (Russo, 2015). In a recent study, the ECG characteristics of such arrhythmia were delineated (Sadek et al., 2015). MB VAs exhibit an LBBB pattern with a left superior axis, a precordial sharp downstroke of the QRS complex, and a relatively narrow QRS width. The precordial transition is typically after V4 and always later than that of the sinus QRS (Sadek et al., 2015). The late precordial transition and superiorly directed axis help to differentiate MB VAs from the VAs originating in the RV base or septum, whereas no identified ECG features could clearly differ them from the anterior PAP VAs (Sadek et al., 2015; Van Herendael et al., 2011). It is noteworthy that PVCs originating from the MB could trigger idiopathic ventricular fibrillation and was previously under‐recognized (Haissaguerre et al., 2002; Russo, 2015).

#### 4.2.5 The parahisian region

The parahisian region is an unneglectable site for the IVAs because of its complex anatomical relationship with adjacent structures. The parahisian region locates near yet more rightward, inferior, and posterior to the RVOT. Therefore, VAs originating near the His‐bundle show an LBBB and left inferior axis morphology with monophasic tall R waves in lead I (Yamada, McElderry, Doppalapudi, & Kay, 2008). More specifically, an R wave in lead aVL, along with QS pattern in lead V1 and an early precordial transition (V2‐V3) could be seen in most cases (Yamauchi et al., 2005). The index of the R‐wave ratio (R III/R II) significantly differs the parahisian VAs from the RVOT VAs because of the left directed axis (smaller R wave in leads III and aVF but not in lead II). Moreover, the QRS duration in leads II, III, and aVF is significantly narrower and the R‐wave amplitude in leads V5 and V6 is much greater in the VAs originating near the His‐bundle. The immediate vicinity of the His‐bundle that causes excitement penetrating into the His‐Purkinje system might explain the discrepancies. According to the study by Komatsu et al. (2012), parahisian VAs could be classified into two subgroups, either above or below the HB region. The “above” group remains some parahisian ECG characteristics (narrower QRS duration in the inferior leads, R wave in lead aVL) but mimics the RVOT VAs even more (smaller R wave in lead I, greater R‐wave ratio).

#### 4.2.6 Others

IVA originating from the junction between RVOT and TA (RTJ) is a distinct subgroup of right ventricular IVAs (Lu et al., 2016). The QRS pattern in lead aVL shows a flat morphology in RTJ VAs compared with a deep negative pattern in RVOT VAs and a tall positive pattern in TV VAs because of its intermediate position. IVAs originating from the RTJ show a positive QRS polarity in lead I, II, III, and aVF (Lu et al., 2016). Although parahisian IVAs appear similar ECG features with a flat QRS in lead aVL (Letsas, Efremidis, Tsikrikas, & Sideris, 2013), a narrower QRS duration might differentiate the parahisian IVAs from the RTJ IVAs.

The right ventricular apex is an extremely rare site of origin with few reports (Letsas et al., 2013; Navarrete, 2008). The ECG patterns are characterized by late precordial transition (≥V6), negative QRS complexes in all inferior leads, smaller R wave in lead II and S wave in lead aVR (Van Herendael et al., 2011).

### 4.3 Left Ventricle

The aortic root is the most common site of origin in the left ventricle(70%), followed by the LV summit(12%) and the LV ostium(about 5%–10%)(Wasmer et al., 2013; Yamada, McElderry, Doppalapudi, & Kay, 2008). Other sites of origin such as papillary muscles, fascicles, and cardiac crux are less frequent Ouyang et al., 2014).

#### 4.3.1 LVOT

Because of their close localization with RVOT (Asirvatham, 2009), IVAs originating from the aortic sinus of Valsalva(ASV) represent an LBBB and inferior axis morphology which mimics the RVOT VAs with rare exception. They usually demonstrate an earlier precordial transition with broader and taller R waves in lead V1 and V2 (Hachiya et al., 2002). When the precordial transition is later than lead V4, the prior consideration should be the RVOT VA. However, it is difficult to distinguish the RVOT VAs and the ASV VAs when the precordial transition is in lead V3 (Yamada, 2016). Some ECG algorithms might be recommended under such circumstance including indexes of R‐wave duration and R/S‐wave amplitude ratio in lead V1 or V2, V2 transition ratio and the V2S:V3R index (Betensky et al., 2011; Y. Wang, Liang, Wu, Han, & Ren, 2018; Yoshida et al., 2014). The RCC locates so close to the RVOT that most ECG parameters except for the precordial transitional zone (either V1 or V2) and the higher R‐wave amplitude in lead I are inefficient to distinguish RCC VAs from RVOT VAs (Hachiya et al., 2002). LCC VAs are characterized by a significant R‐wave or a multiphasic pattern(an “m” or “w” morphology) in lead V1, higher R‐wave amplitude in the inferior leads and lead I and greater R‐wave amplitude ratio (III/II > 0.9) when compared with RCC VA (Lin et al., 2008; Ouyang et al., 2002; Yamada, McElderry, Doppalapudi, Murakami, et al., 2008). This might be explained by the rightward inclination of the aortic valve from the horizontal plane, which gives the LCC a more lateral and superior location to the RCC. The RCC‐LCC commissure is a common origin of IVAs and appears a QS morphology in lead V1 with downward notchings and precordial transition in lead V3 (Bala et al., 2010; Yamada, Yoshida, et al., 2008). NCC VA is an extremely rare type and shows similar ECG features to those of RCC VAs. Narrower QRS duration and smaller III/II ratio indicate the NCC VAs (Yamada et al., 2013). It should be noted that no significant differences could differs parahisian VAs from the RCC VAs nor the NCC VAs (Yamada, McElderry, Doppalapudi, & Kay, 2008).

IVAs originating from AMC exhibit an LBBB QRS pattern and a qR pattern in lead V1 resulting from its unique location which leads to primordial septal activation followed by rapid lateral to medial basal LV activation (Dixit et al., 2005). However, AMC VAs may also display an RBBB pattern without S wave in almost all the precordial leads or other QRS morphologies (Chen et al., 2012; Kumagai et al., 2008). Kumagai et al. also found that both AMC VAs and mitral annulus VAs have longer intrinsicoid deflection time (IDT) than the ASV VAs.

The septal‐parahisian sites refer to LV septum underneath the aortic valve in close vicinity to the His‐bundle and membranous septum and, therefore, its ECG characteristics are similar to those of RCC (Yamada, Plumb, et al., 2010). Specifically, IVAs from this region display an LBBB configuration with left inferior axis and relatively early precordial transitional zone in lead V2‐V3 (Park et al., 2012). Yamada et al. reported that they could be differentiated by a negative QRS polarity in lead III and by a positive QRS polarity in lead aVL (Yamada, Plumb, et al., 2010).

#### 4.3.2 Epicardial Regions

An epicardial origin could be found in about 15% of outflow tract arrhythmias, among which the LV summit is the most common site of origin with an inaccessible area for ablation (Baman et al., 2010). The MDI (maximum deflection index) might be useful to distinguish the epicardial IVAs (>0.55) from the endocardial IVAs (Daniels et al., 2006). This index, however, is not eternally reliable (Baman et al., 2010; Yamada, Mcelderry, et al., 2010). There are other parameters supporting an epicardial origin such as a pseudodelta wave (≥34 ms), an intrisicoid deflection time (≥85 ms), and an RS complex duration (≥121 ms) (Berruezo et al., 2004). The superior portion of this region is not accessible for catheter ablation. An RBBB pattern, transitional zone earlier than lead V1, aVL/aVR amplitude ratio > 1.1, and presence of S waves in lead V5 or V6 could be seen as the criteria to predict the ablation feasibility within the area (Yamada, McElderry, et al., 2010).

All crux IVAs have a superior axis and QS wave in inferior leads and mostly have prominent R wave in lead V2. Apical crux IVAs display an RBBB or LBBB pattern with positive QRS complex in lead aVR and negative QRS in lead V6 (Kawamura et al., 2014, 2015). Basal crux IVAs exhibit an LBBB morphology with an early transition in lead V2.

#### 4.3.3 Mitral Annulus

About 5% IVAs stem from the MA with distinctive ECG characteristics (Wasmer et al., 2013). Generally, they represent an RBBB pattern with early precordial R‐wave transition (usually in lead V1, some between lead V1 and V2), a concordant positive QRS pattern in leads V2‐V4 and an S wave in lead V6 (Kumagai et al., 2005; Tada et al., 2005). These might be explained by the fact that the MA is located in the posterior portion of the LV, distant from the precordial electrodes resulting in a depolarization direction toward these electrodes (Tada et al., 2005). However, to identify more detailed sites of origin, more criteria are needed. (Tada et al., 2005) recommended that polarity of the QRS complex in the inferior leads and leads I and aVL, negative component of the QRS complex in leads I and V1, “notching” of the late phase of the QRS complex in the inferior leads, and a longer QRS duration could help confirm the precise origin at the MA. The “notching” could not only be observed in anterolateral MA IVAs and posterior MA IVAs, namely the free‐wall of the LV, but also in the RVOT free‐wall IVAs. Phased excitation from the free wall to the other ventricle might provide basis for the phenomenon (Tada et al., 2005). Kumagai et al. also introduced a tactic to identify the precise origin by using S wave in lead V6, IDT, and R‐wave polarity in inferior leads (Kumagai et al., 2005).

#### 4.3.4 Left papillary muscles

The anterior and posterior papillary muscles(APM and PPM) in the left ventricle are connected respectively with the anterior and posterior leaflet of the mitral valve. IVAs originate predominantly from the posterior papillary muscle (Naksuk et al., 2016). Because the LV papillary muscles are in close relationship with the Purkinje fiber, similar to the moderator band, the site of origin of PPM arrhythmias was often found to be the Purkinje fiber–muscular interface (Good et al., 2008). Thus, it is difficult to distinguish the two arrhythmias. Both APM and PPM VAs exhibit an RBBB morphology with an early precordial transition before lead V1. Arrhythmias from the PPM usually have (left or right) superior axis like arrhythmias from the left posterior fascicle and posterior half of the mitral annulus. Similarly, arrhythmias from the APM, the left anterior fascicle and the anterior half of the mitral annulus often have inferior axis (Al'Aref et al., 2015; Doppalapudi et al., 2008; Yamada et al., 2009). To differentiate arrhythmias from PAP, left fascicles, and mitral annulus, a stepwise algorithm was proposed with relatively high sensitivity and specificity using parameters such as frontal plane axis, QRS duration, precordial transition, and lead V1 morphology(Al'Aref et al., 2015). Arrhythmias from the left PAP display longer QRS duration than that of fascicular VAs, often no positive precordial concordance and no presence of V1 r < R’.

#### 4.3.5 Left Fascicles

The left fascicular VT, also called verapamil‐sensitive VT, could be divided into three subtypes due to involved fascicle (Nogami, 2011). The left posterior fascicular VT is most commonly seen (up to 90%) with an RBBB configuration QRS complex, a superior axis and RS complexes in lead V5 and V6. The left anterior fascicular form is uncommon (approximately 10%), whose QRS morphology exhibits an RBBB configuration and right‐axis deviation. The left upper septal fascicular form is rarely seen (less than 1%) and is remarkable for its narrow QRS configuration with normal or right‐axis deviation (Guo et al., 2018; Haqqani et al., 2009).

## 5 TREATMENT OF IVAS

Since catheter ablation emerged, accurate localization of IVAs became much more required. Therefore, surface ECG is extremely important to help better diagnosis and treatment. The most common indication for treating PVCs without structural heart disease (SHD) remains the presence of symptoms that are not improved by an explanation of their benign nature and reassurance from the physician. However, it has been validated that there exists a potential association between frequent PVCs burden and cardiomyopathy which is treatable by catheter ablation (Bogun et al., 2007; Chugh, Shen, Luria, & Smith, 2000; Yarlagadda et al., 2005). A clear‐cut point at which LV function impairment is unavoidable is undetermined. The percentage of PVCs per day associated with LV function impairment has generally been reported at burdens above 15%–25% of total beats (Pedersen et al., 2014). Nevertheless, the majority of patients with frequent PVCs have a benign course whereas up to one third of them develop cardiomyopathy (Lee, Klarich, Grogan, & Cha, 2012). Therefore, other factors such as longer PVC durations, presence of nonsustained VT, multiform PVCs, and retrograde P waves may also play a role in this process (Ban et al., 2013; Del Carpio Munoz et al., 2011). For patients with > 10 000 PVCs/24h, according to an expert consensus, follow‐up with repeat echocardiography and Holter Monitoring should be considered. Catheter ablation of PVCs is recommended for highly selected patients who remain symptomatic despite conservative treatment or for those with high PVC burdens associated with a decline in the LV systolic function (Pedersen et al., 2014).

Idiopathic VTs are basically monomorphic and hemodynamically stable (Yamada, 2016). Most idiopathic nonsustained VTs originate from the RVOT or LVOT and only require treatment if they are symptomatic, incessant, or produce LV dysfunction. The treatment of these outflow tract VTs is either medical or catheter ablation (Pedersen et al., 2014; Stevenson & Soejima, 2007). In patients with symptomatic, idiopathic, sustained monomorphic VT, catheter ablation might be preferable to medical therapy. It is a more definitive treatment option, given its high success and low recurrence rate (Cronin et al., 2019; Krittayaphong et al., 2006; Miyazawa et al., 2017).

## 6 CONCLUSION

Surface ECG is a useful and convenient tool to identify the site of origin of IVAs, which usually exhibit characteristic ECGs based on their anatomical and mechanical background. Therefore, understanding special anatomical correlations is necessary to distinguish varied sites of origin. Special ECG characteristics of different origins have been summarized above but there remain some overlaps and difficulties for accurate identifications. More highly specific indications and molecular basis for IVAs are needed in the future research.
